# Synthesis of Quinoxaline 1,4-di-*N*-Oxide Analogues and Crystal Structure of 2-Carbomethoxy-3-hydroxyquinoxaline-di-*N*-oxide

**DOI:** 10.3390/molecules16086894

**Published:** 2011-08-12

**Authors:** Yingjun Xu, Fanhong Wu, Zhiyi Yao, Minmin Zhang and Sheng Jiang

**Affiliations:** College of Chemical and Environmental Engineering, Shanghai Institute of Technology, Shanghai 200235, China

**Keywords:** quinoxaline 1,4-di-*N*-oxide, Beirut reaction, acyloin-endiol tautomerism

## Abstract

A series of quinoxaline 1,4-di-*N*-oxide analogues were prepared from benzofurazan *N*-oxide derivatives and β-diketone ester compounds by the improved Beirut reaction. The structures of the target products were characterized by NMR, MS, IR and elemental analysis measurements, and that of 2-carbomethoxy-3-hydroxyquinoxaline- di-N-oxide was further confirmed by single-crystal X-ray diffraction. Its crystal structure belongs to the monoclinic system, space group C2/c with a = 14.4320 (12) Å, b = 10.7514 (9) Å, c = 13.2728 (11) Å, V = 1958.5 (3) Å^ 3^, Z = 8. The X-ray crystallographic analysis reveals that quinoxaline 1,4-di-*N*-oxide displays acyloin-endiol tautomerism.

## 1. Introduction

Some quinoxaline 1,4-di-*N*-oxides and their derivatives are useful materials that possess a wide range of antimicrobial activities and animal growth promotion effects [1,2,3,4]. Benzofurazan *N*-oxide derivatives can react with enamines [5], β-diketone esters [6], or any aldehyde or ketone containing two α-H [7,8] to form quinoxaline 1,4-di-*N*-oxides. This reaction, which has been referred to as the Beirut reaction, is an excellent method for preparing these heterocyclic compounds. The applicable reaction systems include triethylamine [9], triethylamine/methanol [10], and KOH/methanol [11], with long reaction times (8 h-2 d) and 50%–80% yield. When we first set out to discover novel and biologically active compounds, we managed to synthesize a series of quinoxaline 1,4-di-*N*-oxide analogues with the NaH/THF reaction system, which offered shorter reaction times and gave higher yields.

## 2. Results and Discussion

We synthesized a series of quinoxaline 1,4-di-*N*-oxide analogues **1-10** by the NaH/THF reaction system, which allows the reaction times to be shortened to 2–4 h with yields ranging from 60% to 80%. All these analogues are new compounds and have not been previously reported.

**Scheme 1:**
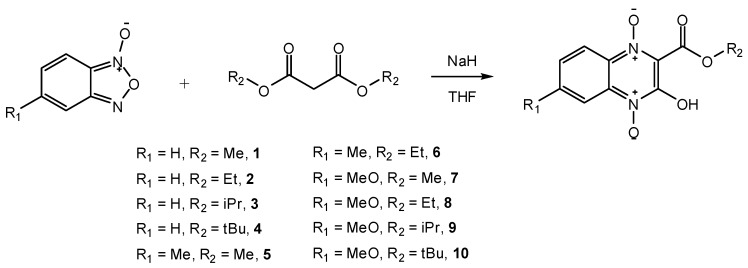
Synthesis of quinoxaline 1,4-di-*N*-oxide analogues.

**Figure 1:**
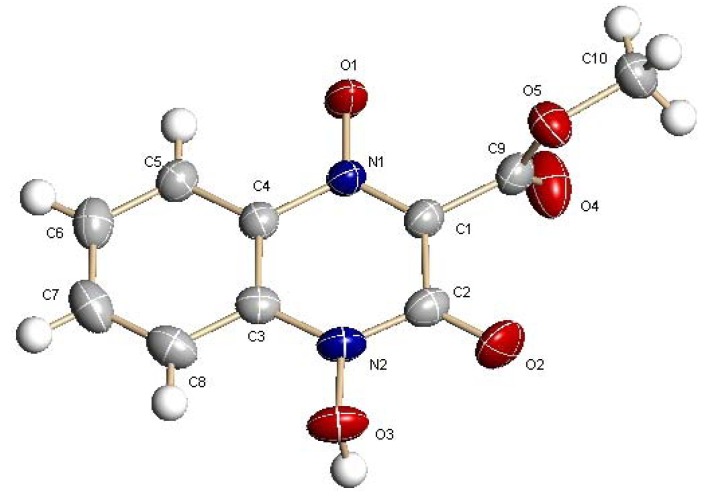
X-Ray crystal structure of **1**.

The configurations of the products were determined by IR, NMR, MS and elemental analysis. By those measurements, it can be clearly confirmed that these molecules had a quinoxaline di-*N*-oxide skeleton and the corresponding side chains such as carboalkyl. And on account of this, we believe that our synthesis method was feasible. However, NMR could not differentiate the absolute configurations of C–OH and C=O. Therefore, to obtain deeper insights into these compounds, the absolute configuration of analog **1** was determined by X-ray diffraction. The geometry of **1** is illustrated in Figure 1, and selected bond lengths are given in Table 1.

**Table 1:** Selected bond lengths (Å) for **1**.

Bond	Bond length
O(1)–N(1)	1.2903 (13)
O(2)–C(2)	1.2115 (17)
O(3)–N(2)	1.3844 (14)

**Scheme 2:**
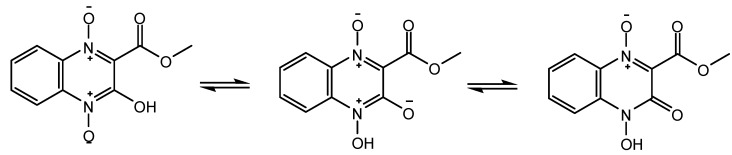
Acyloin-endiol tautomerism of **1**.

The crystal structure belongs to monoclinic system, space group C2/c with a = 14.4320 (12) Å, b = 10.7514 (9) Å, c = 13.2728 (11) Å, V = 1958.5 (3) Å^3^, Z = 8. The X-ray crystallographic analyses revealed that the quinoxaline 1,4-di-*N*-oxide has an acyloin-endiol tautomerism. As shown in Table 1, the O(3)–N(2) bond (1.3844 (14) Å) was longer than the O(1)–N(1) bond (1.2903 (13) Å). As the general length of O^-^–N^+ ^was about 1.29 Å [12], we believed that O(3)–N(2) bond become longer as a result of protolysis. The length of the O(2)–C(2) bond was 1.2115 (17) Å, which is between the general length of the C-O (1.45 Å) and C=O (1.19 Å) bonds and much more close to the C=O length, so we believe the O(2)–C(2) bond was a C=O bone. As shown in Scheme 2, we hold that there is an acyloin-endiol tautomerism, and as the keto form can provide a conjugate structure, it is more stable than the enol form. 

## 3. Experimental

### 3.1. General

All reagents were of high purity, purchased from either Aldrich or TCI, and used without further purification. Analytical grade tetrahydrofuran, dichloromethane, methanol, ethyl acetate, and petroleum ether were obtained from Shanghai Chemicals, China. Yields were not optimized. Melting points were measured in capillary tubes using a WRS-1B apparatus and remained uncorrected. Elemental analyses were performed by a Vario EL III elemental analyser. Nuclear magnetic resonance (NMR) spectra were determined in methanol-d_4_ or CDCl_3_ solutions using a Bruker 500 MHz spectrometer (operating at 500 MHz for ^1^H and 125.75 MHz for ^13^C). Chemical shifts are reported in parts per million (ppm, δ) downfield from tetramethylsilane. Splitting patterns are described as singlet (s), doublet (d), triplet (t), quartet (q), multiplet (m), and broad (br). Infrared (IR) spectra were determined using a Bruker IFS-48 FT-IR IR spectrometer. Window material was prepared with potassium bromide (KBr) powder, and the results are reported in wavenumbers (cm^−1^). Mass spectra (MS) were obtained with electric or chemical ionization (ESI) on a MAT-95 spectrometer.

### 3.2. General Synthesis Procedure

β-Diketone ester (1.6 mmol) was added with stirring to a solution of NaH (0.0384 g, 1.6 mmol) in anhydrous THF (8 mL) at 0–5 °C. The reaction mixture was stirred for 0.5 h at room temperature and then cooled to 0–5 °C. A solution of benzofuroxan *N*-oxide (1.5 mmol) in anhydrous THF (3 mL) was slowly added to the foregoing mixture. The reaction mixture was stirred for the indicated period of time at room temperature. The resulting solid product was filtered off, dissolved in water, adjusted to acidic, and then extracted with chloroform. The final organic layer was dried under anhydrous Na_2_SO_4_, and the solvent was removed by evaporation under vacuum to obtain the products.

*Synthesis of 2-carbomethoxy-3-hydroxyquinoxaline-di-N-oxide* (**1**). Following the general procedure, dimethyl malonate (0.1836 mL, 1.6 mmol) was allowed to react with benzofuroxan *N*-oxide (0.2042 g, 1.5 mmol) for 2 h. The product **1** was obtained as brown crystals (0.2783 g, yield 78.6%). M.p. = 161.0–161.2 °C (dec.). IR (KBr): 3446 (N-O-H), 2914 (C-H), 1742 (C=O), 1613 and 1529 (aromatic ring stretch), 1096 (C-N), 966 (N-O), 771 and 745 cm^−1^ (ring C-H deformation). ^1^H-NMR (methanol-d_4_): 8.33 (d, *J* = 10.0 Hz, 1H), 7.93–7.86 (m, 2H), 7.52 (t, *J* = 15.0 Hz, 1H), 4.02 (s, 3H). ^13^C-NMR (methanol-d_4_): 159.8, 151.2, 133.9, 133.1, 132.6, 129.9, 124.6, 119.7, 113.6, 52.6. Anal. calcd for C_10_H_8_N_2_O_5_: C, 50.85; H, 3.41; N, 11.86. Found: C, 51.83; H, 3.62; N, 11.89. LC-MS: *m*/*z* Calcd [M^+^]: 236.0. Found [M^+^]: 236.0.

*Synthesis of 2-carboethoxy-3-hydroxyquinoxaline-di-N-oxide* (**2**). Following the general procedure, diethyl malonate (0.2418 mL, 1.6 mmol) was allowed to react with benzofuroxan *N*-oxide (0.2042 g, 1.5 mmol) for 4 h. The pale yellow solid **2** was recrystallized from CH_2_Cl_2_ (0.2398 g, yield 63.9%). M.p.=167.5–168.2 °C (dec.). IR (KBr): 3440 (N-O-H), 1743 (C=O), 1629 and 1590 (aromatic ring stretch), 1244 (C-O-C), 1097 (C-N), 1021 (N-O), 773 and 745 cm^−1^ (ring C-H deformation). ^1^H-NMR (methanol-d_4_): 8.33 (d, *J =* 5.0 Hz, 1H), 7.93–7.86 (m, 2H), 7.53 (t, *J =* 15.0 Hz, 1H), 4.50 (q, *J =* 20.0 Hz, 2H), 1.42 (t, *J =* 15.0 Hz, 3H). ^13^C-NMR (methanol-d_4_): 159.3, 151.2, 133.8, 133.1, 132.7, 129.9, 124.6, 119.7, 113.6, 62.7, 12.9. Anal. calcd for C_11_H_10_N_2_O_5_: C, 52.80; H, 4.03; N, 11.20. Found: C, 53.64; H, 4.12; N, 11.21. LC-MS: *m*/*z* Calcd [M^+^]: 250.1. Found: 250.9.

*Synthesis of 2-carboisopropoxy-3-hydroxyquinoxaline-di-N-oxide* (**3**). Following the general procedure, diisopropyl malonate (0.3039 mL, 1.6 mmol) was allowed to react with benzofuroxan *N*-oxide (0.2042 g, 1.5 mmol) for 2 h. The product **3** was obtained as yellow crystals (0.3121 g, yield 83.2%). M.p. = 177.4–178.7 °C (dec.). IR (KBr): 3446 (N-O-H), 2983 (C-H), 1735 (C=O), 1625 and 1530 (aromatic ring stretch), 1259 (C-O-C), 1095 (C-N), 1019 (N-O), 763 and 737 cm^−1^ (ring C-H deformation). ^1^H- NMR (CDCl_3_): 8.38 (d, *J =* 10.0 Hz, 1H), 7.98 (d, *J =* 10.0 Hz, 1H), 7.82 (t, *J =* 15.0 Hz, 1H), 7.52 (d, *J =* 10.0 Hz, 1H), 5.39–5.32 (m, 1H), 1.39 (d, *J =* 5.0 Hz, 6H). ^13^C-NMR (CDCl_3_): 158.3, 151.5, 134.0, 131.9, 131.3, 130.5, 125.5, 120.5, 114.3, 71.7, 21.6 (2C). Anal. calcd for C_12_H_12_N_2_O_5_: C, 54.55; H, 4.58; N, 10.60. Found: C, 55.36; H, 4.71; N, 10.48; O, 29.45. LC-MS: *m*/*z* Calcd [M^+^]: 264.1. Found: 264.1.

*Synthesis of 2-carbo-tert-butoxy-3-hydroxyquinoxaline-di-N-oxide* (**4**). Following the general procedure, ditertbutyl malonate (0.3582 mL, 1.6 mmol) was allowed to react with benzofuroxan *N*-oxide (0.2042 g, 1.5 mmol) for 2 h. The product **4** was obtained as yellow crystals (0.3024 g, yield 72.5%). M.p. = 161.0–161.1 °C (dec.). IR (KBr): 3445 (N-O-H), 2983 (C-H), 1735 (C=O), 1623 and 1532 (aromatic ring stretch), 1259 (C-O-C), 1098 (C-N), 791 and 755 cm^−1^ (ring C-H deformation). ^1^H-NMR (methanol-d_4_): 8.32 (d, *J =* 5.0 Hz, 1H), 7.92–7.85 (m, 2H), 7.52 (t, *J =* 15.0 Hz, 1H), 1.64 (s, 9H). ^13^C-NMR (methanol-d_4_): 158.3, 151.2, 133.6, 133.0, 129.8, 124.5 (2C), 119.7, 113.6, 85.0, 26.9 (3C). Anal. calcd for C_13_H_14_N_2_O_5_: C, 56.11; H, 5.07; N, 10.07. Found: C, 56.07; H, 5.11; N, 9.74. LC-MS: *m*/*z* Calcd [M^+^]: 278.1. Found: 277.9.

*Synthesis of 2-carbomethoxy-3-hydroxy-6-methylquinoxaline-di-N-oxide* (**5**). Following the general procedure, dimethyl malonate (0.1836 mL, 1.6 mmol) was allowed to react with 5-methyl benzofuroxan *N*-oxide (0.2252 g, 1.5 mmol) for 2 h. The product **5** was obtained as brown crystals (0.2712 g, yield 72.3%). M.p. = 193.2–194.4 °C (dec.). IR (KBr): 3439 (N-O-H), 1750 (C=O), 1616 and 1538 (aromatic ring stretch), 1244 (C-O-C), 1097 (C-N), 753 and 704 cm^−1^ (ring C-H deformation). ^1^H-NMR (methanol-d_4_): 8.13 (s, 1H), 7.80 (d, *J =* 10.0 Hz, 1H), 7.73 (d, *J =* 10.0 Hz, 1H), 4.01 (s, 3H), 2.51 (s, 3H). ^13^C-NMR (methanol-d_4_): 159.9, 151.0, 135.7, 135.2, 132.6, 131.1, 129.7, 119.1, 113.6, 52.5, 19.5. Anal. calcd for C_11_H_10_N_2_O_5_: C, 52.80; H, 4.03; N, 11.20. Found: C, 53.61; H, 4.16; N, 11.25. LC-MS: *m*/*z* Calcd [M^+^]: 250.1. Found: 250.8.

*Synthesis of 2-carboethoxy-3-hydroxy-6-methylquinoxaline-di-N-oxide* (**6**). Following the general procedure, diethyl benzylmalonate (0.2418 mL, 1.6 mmol) was allowed to react with 5-methyl benzofuroxan *N*-oxide (0.2252 g, 1.5 mmol) for 4 h. The pale yellow solid **6** was recrystallized from CH_2_Cl_2_ (0.2408 g, yield 60.8%). M.p. = 208.7–209.7 °C (dec.). IR (KBr): 3440 (N-O-H), 2926 (C-H), 1749 (C=O), 1639 and 1538 (aromatic ring stretch), 1246 (C-O-C), 1098 (C-N), 756 and 705 cm^−1^ (ring C-H deformation). ^1^H-NMR (methanol-d_4_): 8.14 (s, 1H), 7.83 (d, *J =* 10.0 Hz, 2H), 7.73 (d, *J =* 10.0 Hz, 1H), 4.48 (q, *J =* 20.0 Hz, 2H), 2.51 (s, 3H), 1.41 (t, *J =* 15.0 Hz, 3H). ^13^C-NMR (methanol-d_4_): 159.4, 151.1, 135.6, 135.1, 131.1, 129.7, 119.6, 119.0, 113.6, 62.7, 19.5, 12.9. Anal. calcd for C_12_H_12_N_2_O_5_: C, 54.55; H, 4.58; N, 10.60. Found: C, 53.64; H, 4.51; N, 10.37. LC-MS: *m*/*z* Calcd [M^+^]: 264.1. Found: 264.9.

*Synthesis of 2-carbomethoxy-3-hydroxy-6-methoxylquinoxaline-di-N-oxide* (**7**). Following the general procedure, dimethyl malonate (0.1836 mL, 1.6 mmol) was allowed to react with 5-methoxy benzofuroxan *N*-oxide (0.2492 g, 1.5 mmol) for 2 h. The product **7** was given as brown crystals (0.3242 g, yield 81.2%). M.p. = 168.7–170.4 °C (dec.). IR (KBr): 3448 (N-O-H), 2954 (C-H), 1744 (C=O), 1616 and 1527 (aromatic ring stretch), 1250 (C-O-C), 1015 (C-N), 827 and 763 cm^−1^ (ring C-H deformation). ^1^H-NMR (CDCl_3_): 7.90 (s, 1H), 7.61 (d, *J =* 10.0 Hz, 1H), 6.96 (t, *J =* 29.0 Hz, 1H), 3.96 (s, 3H), 3.87 (s, 3H). ^13^C-NMR (CDCl_3_): 159.5, 157.9, 150.5, 131.8, 131.3, 126.0, 124.7, 115.8, 101.0, 55.9, 53.7. Anal. calcd for C_12_H_12_N_2_O_5_: C, 49.63; H, 3.79; N, 10.52. Found: C, 49.58; H, 4.10; N, 10.47. LC-MS: *m*/*z* Calcd [M^+^]: 266.1. Found: 266.9.

*Synthesis of 2-carboethoxy-3-hydroxy-6-methoxylquinoxaline-di-N-oxide* (**8**). Following the general procedure, diethyl malonate (0.2418 mL, 1.6 mmol) was allowed to react with 5-methoxy benzofuroxan *N*-oxide (0.2492 g, 1.5 mmol) for 4 h. The pale yellow solid **8** was recrystallized from CH_2_Cl_2_ (0.2849 g, yield 67.8%). M.p. = 201.5–201.8 °C (dec.). IR (KBr): 3441 (N-O-H), 2982 (C-H), 1739 (C=O), 1617 and 1527 (aromatic ring stretch), 1248 (C-O-C), 1014 (C-N), 829 and 767 cm^−1^ (ring C-H deformation). ^1^H-NMR (CDCl_3_): 7.89 (d, *J =* 10.0 Hz, 1H), 7.82 (d, *J =* 10.0 Hz, 1H), 7.46 (q, *J =* 15.0 Hz, 1H), 4.54 (q, *J =* 20.0 Hz, 2H), 3.94 (s, 3H), 1.43 (t, *J =* 15.0 Hz, 3H). ^13^C-NMR (CDCl_3_): 158.9, 157.8, 150.0, 131.8, 131.2, 125.3, 124.7, 115.4, 101.1, 63.3, 56.2, 14.0. Anal. calcd for C_12_H_12_N_2_O_6_: C, 51.43; H, 4.32; N, 10.00. Found: C, 51.27; H, 4.31; N, 9.91. LC-MS: *m*/*z* Calcd [M^+^]: 280.1. Found: 280.9.

*Synthesis of 2-carboisopropoxy-3-hydroxy-6-methoxylquinoxaline-di-N-oxide* (**9**). Following the general procedure, diisopropyl malonate (0.3039 mL, 1.6 mmol) was allowed to react with 5-methoxy benzofuroxan *N*-oxide (0.2492 g, 1.5 mmol) for 2 h. The product **9** was given as yellow crystals (0.3313 g, yield 75.1%). M.p. = 212.0–212.5 °C (dec.). IR (KBr): 3439 (N-O-H), 2982 (C-H), 1742 (C=O), 1616 and 1528 (aromatic ring stretch), 1250 (C-O-C), 1103 (C-N), 825 and 765 cm^−1^ (ring C-H deformation). ^1^H-NMR (methanol-d_4_): 7.87 (d, *J =* 5.0 Hz, 1H), 7.78 (s, 1H), 7.51 (d, *J =* 5.0 Hz, 1H), 5.34 (t, *J =* 10.0 Hz, 1H), 3.92 (s, 3H), 1.42 (d, *J =* 5.0 Hz, 6H). ^13^C-NMR (methanol-d_4_): 159.0, 157.5, 150.6, 133.2, 130.6, 127.5, 123.4, 100.8, 71.2, 55.2, 20.4 (2C). Anal. calcd for C_13_H_14_N_2_O_6_: C, 53.06; H, 4.80; N, 9.52. Found: C, 54.01; H, 4.97; N, 9.55. LC-MS: *m*/*z* Calcd [M^+^]: 294.1. Found: 294.0.

*Synthesis of 2-carbotertbutoxy-3-hydroxy-6-methoxylquinoxaline-di-N-oxide* (**10**). Following the general procedure, ditertbutyl malonate (0.3582 mL, 1.6 mmol) was allowed to react with 5-methoxy benzofuroxan *N*-oxide (0.2492 g, 1.5 mmol) for 2 h. The product **10** was given as yellow crystals (0.3291 g, yield 71.2%). M.p. = 261.0–262.5 °C (dec.). IR (KBr): 3442 (N-O-H), 2982 (C-H), 1742 (C=O), 1615 and 1530 (aromatic ring stretch), 1257 (C-O-C), 1013 (C-N), 829 and 786 cm^−1^ (ring C-H deformation). ^1^H-NMR (methanol-d_4_): 7.88 (d, *J =* 10.0 Hz, 1H), 7.79 (s, 1H), 7.51 (t, *J =* 10.0 Hz, 2H), 3.94 (s, 3H), 1.64 (s, 9H). ^13^C-NMR (methanol-d_4_): 158.4, 157.5, 150.6, 133.6, 130.5, 127.4, 123.2, 115.1, 100.8, 85.0, 55.2, 26.9 (3C). Anal. calcd for C_14_H_16_N_2_O_6_: C, 54.54; H, 5.23; N, 9.09. Found: C, 54.18; H, 5.16; N, 8.85. LC-MS: *m*/*z* Calcd [M^+^]: 308.1. Found: 308.0.

### 3.3. Crystal structure determination

Single crystals of **1** suitable for X-ray crystal structure analysis were obtained by growth under slow evaporation at 5 °C from a methanol/water mixture (1:1 v/v). Crystal data and structure solutions at T = 293 (2) K: **1 **C_10_H_8_N_2_O_5_, M_r _= 236.18, monoclinic, C2/c, a = 14.4320 (12) Å, b = 10.7514 (9) Å, c = 13.2728 (11) Å, V = 1958.5 (3) Å^3^, Z = 8, D_x _= 1.602 Mg/m^3^, F(000) = 976, λ(MoKα) = 0.71073 Å, μ = 0.131 mm^−1^. The intensity data were collected on a Bruker Smart CCD diffractometer with graphite monochromated MoKα radiation and the ω-2θ scan technique [2θmax = 54.00°]. The structures were determined by direct methods using SHELXS-97 [13] and expanded using Fourier techniques [14]. The non-hydrogen atoms were refined anisotropically. The final cycle of full-matrix least-squares refinement was based on 1859 observed reflections [I > 2σ(I)] and 160 variable parameters and converged with unweighted and weighted factors of **1 **(R_1 _= 0.0439 and R_w2 _= 0.1220). Neutral atom scattering factors were taken from Cromer and Waber [15]. Anomalous dispersion effects were included in F_calc _ [16]. The values used for Δf´ and Δf″ were those of Creagh and McAuley [17], while the values for the mass attenuation coefficients were those of Creagh and Hubbl [18]. All calculations were performed using the teXsan [19] crystallographic software from Molecular Structure Corporation.

## 4. Supplementary Material

Supplementary crystallographic data have been deposited with the Cambridge Crystallographic Data Centre, CCDC No. 830377. Copies of this information may be obtained free of charge from The Director, CCDC, 12 Union Road, Cambridge CB2 1EZ, UK (Fax: +44 1223 336033; Email: deposit@ccdc.cam.ac.uk or www: http://www.ccdc.cam.ac.uk).

## 5. Conclusions

In summary, we have reported here the synthesis of a series of quinoxaline 1,4-di-*N*-oxide analogues from benzofurazan *N*-oxide derivatives and β-diketone ester compounds by an improved Beirut reaction. The structures of the target products were characterized by NMR, MS, IR and elemental analysis measurements, and the structure of 2-carbomethoxy-3-hydroxyquinoxaline- di-N-oxide (**1**) was further confirmed by single-crystal X-ray diffraction. The X-ray crystallographic analysis reveals that the quinoxaline 1,4-di-*N*-oxide moiety displays acyloin-endiol tautomerism.
